# Melatonin Suppresses Macrophage M1 Polarization and ROS-Mediated Pyroptosis via Activating ApoE/LDLR Pathway in Influenza A-Induced Acute Lung Injury

**DOI:** 10.1155/2022/2520348

**Published:** 2022-11-15

**Authors:** Meng-Meng Xu, Jia-Ying Kang, Shuang Ji, Yuan-Yuan Wei, Si-Liang Wei, Jing-Jing Ye, Yue-Guo Wang, Ji-Long Shen, Hui-Mei Wu, Guang-He Fei

**Affiliations:** ^1^Department of Respiratory and Critical Care Medicine, The First Affiliated Hospital of Anhui Medical University, Hefei, 230022 Anhui, China; ^2^Key Laboratory of Respiratory Disease Research and Medical Transformation of Anhui Province, The First Affiliated Hospital of Anhui Medical University, Hefei, 230022 Anhui, China; ^3^Department of Emergency Critical Care Medicine, First Affiliated Hospital of Anhui Provincial Hospital, Division of Life Science and Medicine, University of Science and Technology of China, Hefei, 230001 Anhui, China; ^4^Provincial Laboratory of Microbiology and Parasitology of Anhui Medical University, Hefei, 230022 Anhui, China; ^5^Anhui Geriatric Institute, Department of Geriatric Respiratory Critical and Care Medicine, The First Affiliated Hospital of Anhui Medical University, Hefei, 230022 Anhui, China

## Abstract

Influenza virus infection is one of the strongest pathogenic factors for the development of acute lung injury (ALI)/ acute respiratory distress syndrome (ARDS). However, the underlying cellular and molecular mechanisms have not been clarified. In this study, we aim to investigate whether melatonin modulates macrophage polarization, oxidative stress, and pyroptosis via activating Apolipoprotein E/low-density lipoprotein receptor (ApoE/LDLR) pathway in influenza A-induced ALI. Here, wild-type (WT) and ApoE-/- mice were instilled intratracheally with influenza A (H3N2) and injected intraperitoneally with melatonin for 7 consecutive days. In vitro, WT and ApoE-/- murine bone marrow-derived macrophages (BMDMs) were pretreated with melatonin before H3N2 stimulation. The results showed that melatonin administration significantly attenuated H3N2-induced pulmonary damage, leukocyte infiltration, and edema; decreased the expression of proinflammatory M1 markers; enhanced anti-inflammatory M2 markers; and switched the polarization of alveolar macrophages (AMs) from M1 to M2 phenotype. Additionally, melatonin inhibited reactive oxygen species- (ROS-) mediated pyroptosis shown by downregulation of malonaldehyde (MDA) and ROS levels as well as inhibition of the NLRP3/GSDMD pathway and lactate dehydrogenase (LDH) release. Strikingly, the ApoE/LDLR pathway was activated when melatonin was applied in H3N2-infected macrophages and mice. ApoE knockout mostly abrogated the protective impacts of melatonin on H3N2-induced ALI and its regulatory ability on macrophage polarization, oxidative stress, and pyroptosis. Furthermore, recombinant ApoE3 (re-ApoE3) inhibited H3N2-induced M1 polarization of BMDMs with upregulation of MT1 and MT2 expression, but re-ApoE2 and re-ApoE4 failed to do this. Melatonin combined with re-ApoE3 played more beneficial protective effects on modulating macrophage polarization, oxidative stress, and pyroptosis in H3N2-infected ApoE-/- BMDMs. Our study indicated that melatonin attenuated influenza A- (H3N2-) induced ALI by inhibiting the M1 polarization of pulmonary macrophages and ROS-mediated pyroptosis via activating the ApoE/LDLR pathway. This study suggested that melatonin-ApoE/LDLR axis may serve as a novel therapeutic strategy for influenza virus-induced ALI.

## 1. Background

Acute lung injury (ALI)/acute respiratory distress syndrome (ARDS) is a rapidly progressing and refractory disease. Particularly, seasonal influenza-induced ALI/ARDS increasingly contributes to annual global mortality. Recent studies indicated that seasonal influenza epidemics annually affected 10–30% of the human population and cause 3-5 million severe cases and approximately 290,000-650,000 deaths worldwide especially among the elderly with chronic diseases [1, 2]. And it was estimated that an annual average of 88,100 influenza-associated respiratory deaths occurred in China [3]. Studies found that influenza viruses A- (H3N2-) associated hospitalizations and mortality were the highest among other circulating viruses [4–6], because it was easier to progress to severe pneumonia with bilateral pulmonary infiltrates, even consolidation (i.e., “white lung” in imageology), and cause ALI/ARDS [1, 3]. Therefore, the further study on cellular and molecular mechanisms of influenza-associated ALI/ARDS remains urgently needed for more efficient agents and therapeutic strategies.

Persistent influenza virus infection leads to robust oxidative stress and cytokine storms (i.e., hypercytokinemia) with extensive pulmonary leukocyte infiltration, edema, and alveolar haemorrhages [7], which are commonly caused by pulmonary immune cells. Therein, macrophages are the most important innate immune cells and constitute the first line of defense against virus and bacteria in ALI/ARDS [8]. As highly plastic cells, macrophages can be polarized into different functional phenotypes, mainly including classically activated (M1) macrophages and alternatively activated (M2) macrophages, which perform different biological functions [9]. In the acute stage of influenza infection, macrophages are polarized into M1 phenotype and release abundant proinflammatory proteins and reactive oxygen species (ROS) [10]. Studies indicated that ROS released by M1 macrophages were prone to causing pyroptosis via activating the NLR family pyrin domain containing 3 (NLRP3) /gasdermin D (GSDMD) pathway [11–13]. Additionally, macrophage polarization may be regulated by Apolipoprotein E (ApoE) signaling [14], and the ApoE/LDLR pathway was suggested to be a potential signaling pathway involved in anti-inflammation and antioxidation [15]. ApoE is an arginine-rich glycoprotein primarily synthesized in the liver, brain, lung epithelia, and macrophages [15, 16]. Low-density lipoprotein receptor (LDLR), the primary binding receptor of ApoE, can assist ApoE to mediate lipid metabolism and the development of pulmonary diseases [15, 17]. Previous studies demonstrated that ApoE knockout caused more severe airway inflammation and oxidative stress in asthmatic mice, whereas administration of an ApoE mimetic peptide suppressed the negative effects in ApoE-/- asthmatic mice, but no effects in LDLR-/- asthmatic mice [18, 19], indicating that ApoE exerted the anti-inflammation and antioxidation probably in a LDLR-dependent manner.

Melatonin (N-acetyl-5-methoxytryptamine, C_13_H_16_N_2_O_2_), a kind of neuroendocrine hormone, is mainly synthesized and secreted by the pineal gland of brains and exerts effects via its membrane receptors (MT1 and MT2) [20, 21]. Melatonin is also synthesized in respiratory epithelia and bone marrow cells as well as macrophages [22, 23]. It is widely recognized that melatonin is in charge of regulating sleep and circadian rhythm and also plays important roles in anti-inflammation and antioxidation [20]. Recent evidence indicated that melatonin had potential abilities of antivirus infection, including respiratory syncytial virus (RSV), influenza virus, and severe acute respiratory syndrome coronavirus 2 (SARS-CoV-2) [24, 25]. Melatonin may inhibit viral replication, improve mitochondrial metabolism via modulating the circadian rhythm, and restrain the “influenza virus-cytokine-trypsin” cycle via inhibiting NLRP3 inflammasome [25]. Moreover, melatonin is also capable of inhibiting the M1 polarization of macrophages to favor antioxidation and anti-inflammation [26]. However, the protective mechanisms of melatonin in influenza virus-induced ALI/ARDS remain unknown.

Taken together, this study was aimed at investigating whether melatonin inhibited influenza virus-induced ALI/ARDS and its underlying molecular mechanisms, which may provide potential therapeutic approaches and efficient agents for influenza-associated ALI/ARDS.

## 2. Materials and Methods

### 2.1. Experimental Animals

Male C57BL/6 mice and ApoE-/- C57BL/6 mice weighing 22-25 g (age 6-8 weeks) were purchased from the Laboratory Animal Center of Hangzhou Ziyuan (Hangzhou, China, Licence key SCXK2019-0004). Experimental mice were housed in the Laboratory Animal Research Center of Anhui Medical University under standard specific pathogen-free condition with 12 h light/12 h dark cycle at 20 ± 2°C. All animal experimental procedures were carried out according to the protocols approved by the Animal Care and Ethics Committee of Anhui Medical University strictly in accordance with ethical principles (Approval No. 20180430).

### 2.2. IAV/H3N2 Amplification and Plaque Assay

Influenza A/Anhui/1/2017 (H3N2) virus was obtained from Prof. Yan Liu (Department of Microbiology, Anhui Medical University, China) and isolated from the patient in 2017 and used in laboratory studies under approved standard biosafety procedures. The influenza A (H3N2) virus samples were amplified in Madin-Darby canine kidney (MDCK) cells and specific pathogen-free embryonated chicken eggs, and virus titers were assayed by standard plaque assay on MDCK cells according to previous description [27]. MDCK cells were infected with diluted virus samples for 2 h at 37°C. After being washed with PBS, the cultivation was proceeded with 50% 2× DMEM, 50% avecil (2.35%), and N-acetyl trypsin (1.5 *μ*g/ml) for 72 h. Then, cells were stained with naphthalene blue-black, and plaques were counted for the calculation of virus titers. All experiments involved in influenza virus were performed according to the biosafety level two requirements with well-equipped personal protection for all the researchers.

### 2.3. Extraction and Culture of Bone Marrow-Derived Macrophages (BMDMs)

Murine bone marrow-derived macrophages (BMDMs) were extracted from the tibias and femurs of wild-type (WT) mice and ApoE-/- mice (age 6-8 weeks) according to previous description [28]. Cut tibias and femurs were flushed with a 1 ml syringe and centrifuged at room temperature, 1500 rpm for 5 min, to obtain bone marrow cells. Bone marrow cells were cultured in Iscove's modified Dulbecco's medium (IMDM) (Gibco, USA) containing 10% Fetal Bovine Serum (FBS) (Excell Biology, New Zealand), 1% penicillin-streptomycin, and 30 ng/ml macrophage colony-stimulating factor (M-CSF, PeproTech, USA) to stimulate differentiation. On day 3, fresh BMDM medium (half of original volume) was supplemented. On day 7, cells were harvested to assess the purity of BMDMs by flow cytometry analysis and plated at a density of 1 × 10^6^ cells/ml for further experiments.

### 2.4. IAV/H3N2-Induced ALI Model and Melatonin Administration

WT mice and ApoE-/- mice were anesthetized with 1% sodium pentobarbital (50 mg/kg) via intraperitoneal injection to ensure that mice are free from pain for invasive trachea cannula. The model mice were instilled intratracheally with 50 *μ*l H3N2 (100 plaque forming units, PFUs) on day 0 and day 3, while the control mice were instilled with 50 *μ*l PBS. The infection period of H3N2 was seven days. From day 0, mice were injected intraperitoneally with PBS or melatonin (Mel) (30 mg/kg, dissolved in PBS containing 5% DMSO) (C_13_H_16_N_2_O_2_, stated purity ≥ 98%, M5250, Sigma-Aldrich, USA) daily at 18:00 for 7 consecutive days. On the seventh day, the mice were sacrificed for further experimental study.

### 2.5. Serum and BALF Collection and Leukocyte Counting

After complete anesthesia with 0.2 ml 1% sodium pentobarbital (about 100 mg/kg) via intraperitoneal injection, bronchoalveolar lavage was performed with 2 ml sterile PBS via an endotracheal tube. The bronchoalveolar lavage fluids (BALF) were centrifuged at 4°C, 1000 rpm for 10 min. Cell pellets were resuspended in PBS with Red Blood Cell (RBC) lysis buffer (C3702, Beyotime Technology, Shanghai, China) for total leukocyte counting using a hemocytometer. Then, smeared BAL cells were stained with Wright-Giemsa stain solution (Baso Diagnostics Inc, Zhuhai, China) for differential leukocyte counting mainly including neutrophils and macrophages in a double-blind manner as previously described [29]. Blood was drawn from the left heart through a 1 ml syringe and centrifuged at 4°C, 4000 rpm for 10 min, to obtain the serum. Serum and BALF of mice were stored in a -80°C freezer for further cytokine analysis.

### 2.6. Measurement of Serum MDA and ApoE Levels and BALF Cytokines

Malondialdehyde (MDA) contents in serum of mice were measured via a MDA assay kit with Thiobarbituric Acid (TBA) methods (A003-1, Nanjing Jiancheng, Nanjing, China) according to the protocols from the manufacturer.

Serum ApoE levels were detected using an ApoE ELISA kit (Elabscience, Wuhan, China), and the levels of IL-1*β* and TNF-*α* in BALF were also measured with the corresponding ELISA kit (MultiSciences, Hangzhou, China), respectively, following the corresponding instructions from the manufacturers.

### 2.7. Histological Analysis and Lung Wet/Dry Ratio

Mice were euthanatized by an enough dose administration of 1% sodium pentobarbital (100 mg/kg, intraperitoneal), the left lung lobes were dissected without proceeding with bronchoalveolar lavage, fixed with 4% paraformaldehyde, and embedded in paraffin. 4 *μ*m sections were stained with haematoxylin and eosin (H&E) for evaluating the severity of lung injury. The indexes of lung injury were double-blindly calculated according to the scoring system mainly including five histological features: (a) neutrophils in the alveolar space, (b) neutrophils in the interstitial space, (c) hyaline membrane formation, (d) proteinaceous debris filling the airspace, and (e) alveolar septal thickening. Each item was scored 0, 1, or 2 based on the severity of lung injury. The final injury scores were figured up according to the following formula [30]: lung injury scores = [(20 × *a*) + (14 × *b*) + (7 × *c*) + (7 × *d*) + (2 × *e*)]/(number of fields × 100).

The degree of lung edema was estimated according to the wet/dry ratio of lung. After anesthesia with 1% sodium pentobarbital (100 mg/kg, intraperitoneal), whole lung tissues were isolated and weighed as the wet weight of the lung. After oven drying at 60°C for 48 h, lung tissues were secondly weighed as the dry weight of the lung. The weight ratio of the wet and dry (W/D) lung was then calculated.

### 2.8. Immunohistochemistry

Immunohistochemistry staining was performed for characterizing the pulmonary localization and expression of melatonin receptors 1/2 (MT-1/2), inducible nitric oxide synthase (iNOS), and Arginase 1 (Arg1). Briefly, 4 *μ*m lung sections were deparaffinized and incubated with corresponding primary antibodies against MT-1/2 (1:50, sc-398788, Santa Cruz, USA), iNOS (1:200, ab178945, Abcam, Cambridge, UK), and Arg1 (1:50, #93668S, Cell Signaling Technology, USA) overnight at 4°C and horseradish peroxidase- (HRP-) conjugated secondary antibody followed by diaminobenzidine (DAB) liquid. The positive expression location of iNOS and Arg1 mainly focused on the areas of leukocyte infiltration along bronchial and alveolar walls.

### 2.9. Flow Cytometry Analysis of BALF Cells and BMDMs

BAL cells were treated with RBC lysis buffer and stained with the following fluorochrome-conjugated antibodies to screen alveolar macrophages (AMs): CD45 (APC-Cy^TM^7, 561037, BD Biosciences, USA), CD11c (PerCP-Cy^TM^5.5, 561114, BD), and Siglec-F (BV421, 565934, BD). For investigating the effects of melatonin on AM polarization, BALF cells were stained with M1 macrophage marker CD86 (PE, 561963, BD) and M2 marker CD206 (MR6F3 APC, ThermoFisher Scientific, USA). Specifically, before transmembrane protein CD206 stained, BALF cells were fixed and permeabilized for better intracellular staining. The images and data of flow cytometry were collected using LSRFortessa X30 (BD Biosciences, USA).

For determining the purity of matured BMDMs, harvested BMDMs were stained with CD45 (APC-Cy^TM^7, BD), F4/80 (PE, 565410, BD), and CD11b (FITC, 557396, BD) antibodies. The purity of BMDMs was assessed via observing the percentage of CD11b^+^F4/80^+^ population.

### 2.10. IAV/H3N2-Infected Cell Injury Model

Murine Raw264.7 cell lines were provided by the Cell Bank of Shanghai Institutes for Biological Sciences (China Academy of Science, Shanghai, China). Raw264.7 cells were cultured in Dulbecco's modified Eagle's medium (DMEM) (Hyclone, Logan, UT, USA) with 10% FBS (Excell biology) at 37°C under saturated humidity conditions and 5% CO_2_. When growing to 60-70% confluence, cells were pretreated with melatonin (100 *μ*M, 200 *μ*M, and 400 *μ*M) for 3 h. Then, the cells were infected with H3N2 (Multiplicity of Infection, MOI = 2) for 6 h, 12 h, 18 h, and 24 h.

For further verifying the effects of melatonin on macrophage polarization, BMDMs were infected with H3N2 (MOI = 2) for 12 h to stimulate the M1 polarization. In the melatonin intervention group, BMDMs were pretreated with melatonin (400 *μ*M) for 3 h before H3N2 infection. Additionally, BMDMs were also pretreated with recombinant ApoE (re-ApoE2, re-ApoE3, and re-ApoE4) (10 *μ*g/ml, PeproTech) before H3N2 infection.

### 2.11. Detection of Cell Viability

Cell viability of Raw264.7 cells stimulated by influenza A (H3N2) with different multiplicities of infection (MOI) was measured by Cell Counting Kit-8 (C0038, Beyotime Technology). Briefly, cells seeded in 96-well plates were incubated with CCK8 working solution for 2 h at 37°C. The absorbance value at 450 nm (OD_450_) was measured by a microplate reader (BioTek Instruments, Vermont, USA).

### 2.12. Detection of Cell Reactive Oxygen Species (ROS) Levels

Cell ROS levels were detected using 2′,7′-dichlorodihydrofluorescein diacetate (DCFH-DA, D6883, Sigma-Aldrich) according to the manufacturer's instructions. Raw264.7 cells were seeded in 96-well plates with a density of 5 × 10^4^/ml with 6 parallel wells, and BMDMs were seeded in 96-well plates with a density of 20 × 10^4^/ml with 6 parallel wells, incubated overnight at 37C, and then treated with corresponding prevention. After 12 h incubation, cells were stained with 10 *μ*M DCFH-DA at 37°C avoiding light for 30 min. ROS levels were determined by Fluorescence Microscopy (Leica, Germany) or Varioskan Flash (Thermo Scientific, USA) at wavelengths of 488 nm for excitation and 525 nm for emission.

### 2.13. Lactate Dehydrogenase (LDH) Release Assay

LDH release in Raw264.7 cells and BMDMs were detected using LDH cytotoxicity assay kit (C0017, Beyotime Technology) according to the protocols from the manufacturer. The absorbance value at 490 nm (OD_490_) was measured by a microplate reader (BioTek Instruments, Vermont, USA).

### 2.14. Immunofluorescence Staining

Raw264.7 cells and BMDMs were fixed with 4% paraformaldehyde for 15 min, followed by permeabilization for 10 min. After blocking with 5% BSA, the cells were incubated with primary antibodies against iNOS (1 : 200, ab178945, Abcam), Arg1 (1 : 50, #93668S, Cell Signaling Technology), and F4/80 (1 : 50, MAB5580-SP, R&D systems, USA) overnight at 4°C. The next day, corresponding secondary antibodies (1 : 500, Alexa Fluor® 488 goat anti-rabbit IgG and Alexa Fluor® 594 goat anti-mouse IgG, Abcam) were applied, followed by the nuclei staining with 4′,6-diamidino-2-phenylindole (DAPI). Confocal lazer scanning analysis of Raw264.7 cells and BMDMs was performed using a laser confocal microscope (Zeiss LSM880, Carl Zeiss AG, Germany).

### 2.15. Reverse Transcription-Polymerase Chain Reaction (RT-PCR)

Total RNA was isolated with TRIzol reagent (Invitrogen, USA), and reverse transcription was conducted using a HyperScript III RT SuperMix for qPCR with gDNA Remover (EnzyArtisan Biotech, Shanghai, China) according to the manufacturer's instruction. RT-PCR was performed using a 2× S6 Universal SYBR qPCR Mix (EnzyArtisan Biotech). All samples were assayed in triplicate, and the target gene expression was normalized to *β*-actin. Relative mRNA expression was calculated with the 2^-∆∆Ct^ method. The specific primers for *β*-actin, MT1, MT2, ApoE, IL-1*β*, TNF-*α*, monocyte chemoattractant protein 1 (MCP1), Arg1, Fizz1, CD86, and CD206 were generated by EnzyArtisan Biotech. The primer sequences are listed in Supplementary Table S1.

### 2.16. Western Blot Analysis

Total proteins were extracted with RIPA lysis containing protease inhibitors from murine lung tissues, Raw264.7 cells, and BMDMs. And protein samples were separated through 10–13% SDS-PAGE and transferred to PVDF membranes. The membranes were incubated with primary antibodies: iNOS (1 : 2000, ab178945, Abcam), Arg1 (1 : 1000, #93668S, Cell Signaling Technology), NLRP3 (1 : 1000, #13158S, Cell Signaling Technology), Caspase1 (1 : 200, sc-392736, Santa Cruz), MT-1/2 (1 : 200, sc-398788, Santa Cruz), GSDMD (1 : 1000, TA4012, Abmart, Shanghai, China), and *β*-actin (1 : 200, sc-47778, Santa Cruz) overnight at 4°C. Subsequently, the membranes were incubated with HRP-conjugated anti-rabbit or anti-mouse secondary antibodies (1 : 2000, Cell Signaling Technology) for 1 h at room temperature. Then, the blots were visualized by Odyssey infrared imaging system (Tanon, Shanghai, China).

### 2.17. Statistical Analysis

All experiments are randomized and blinded. All results were presented as mean ± standard error of the mean (SEM) from at least three independent samples or biological replicates (*n* ≥ 3). Statistical analysis was performed using GraphPad Prism 9.0 (GraphPad Software, Inc., San Diego, CA). Student's *t* test was performed for comparisons between two different groups. One-way ANOVA with Bonferroni's post hoc test (for equal variance) or Dunnett's T3 post hoc test (for unequal variance) was performed for comparisons among multiple groups. ^∗^*p* < 0.05 was considered statistically significant.

## 3. Results

### 3.1. Melatonin Reversed Influenza A- (H3N2-) Induced Decreased Expression of Melatonin Receptors

**Figure 1:**
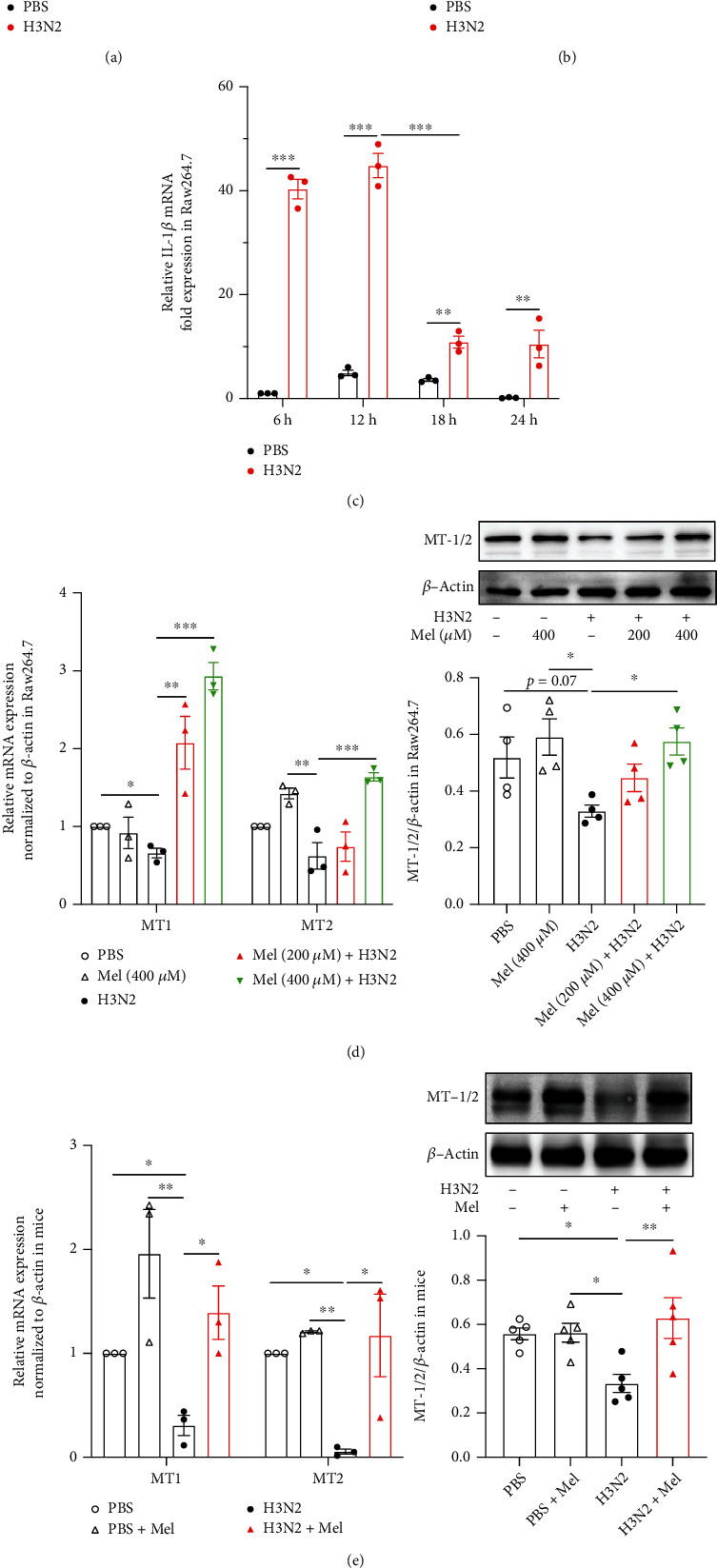
The expression of melatonin receptors in vitro and in vivo. Quantitative reverse transcription-polymerase chain reaction (RT-PCR) measurements of the relative mRNA levels of melatonin receptor 1 (MT1) (a), MT2 (b), and IL-1*β* (c) in Raw264.7 cells infected with influenza A (H3N2) (MOI = 2, 0-24 h). (d) Quantitative RT-PCR measurements and the relative mRNA levels of MT1 and MT2; western blot analysis of the expression of total melatonin receptors (MT-1/2) to *β*-actin in Raw264.7 cells infected with influenza A (H3N2) (MOI = 2, 12 h) with/without melatonin pretreatment (200 *μ*M or 400 *μ*M, 3 h before H3N2 infection). (e) Quantitative RT-PCR measurements of the relative mRNA levels of MT1 and MT2; western blot analysis of the expression of MT-1/2 to *β*-actin in wild-type (WT) mice from the control (PBS) group, PBS+Mel group, H3N2 group, and H3N2+Mel group. (f) Representative immunohistochemical images of lung tissues stained with MT-1/2 as indicated by the brown staining (black arrows) from WT mice, bar 50 *μ*m (original magnification ×50, ×200). Data expressed as mean ± SEM (*n* ≥ 3). ^∗^*p* < 0.05, ^∗∗^*p* < 0.01, and ^∗∗∗^*p*<0.001 compared with influenza A- (H3N2-) infected Raw264.7 cells or mice.

The detection of CCK8 showed that influenza A (H3N2) stimulation with MOI of 2 had no significant effects on the viability of Raw264.7 cells (Figure S1(a)). And H3N2 infection inhibited the mRNA expression of melatonin receptors (MT1 and MT2), which showed a marked decrease after infection at 12 h and recovered at 24 h in Raw264.7 cells (Figures 1(a) and 1(b)). Oppositely, H3N2 infection upregulated IL-1*β* mRNA expression, which reached the peak level at 12 h, then reduced at 18 h and 24 h in Raw264.7 cells (Figure 1(c)). Additionally, in H3N2-infected mouse lung tissues, the mRNA expression of MT1 and MT2 showed decreases compared to the PBS group and PBS+Mel group (Figure 1(e)). However, melatonin intervention significantly increased the mRNA expression of MT1 and MT2 in H3N2-infected Raw264.7 cells and mice (Figures 1(d) and 1(e)). Moreover, the protein expression of MT-1/2 also decreased after H3N2 infection in Raw264.7 cells and mice (Figures 1(d) and 1(e)), suggesting that H3N2 infection may induce a reduced secretion of melatonin. After melatonin treatment, MT-1/2 expression significantly increased in H3N2-infected Raw264.7 cells and mice (Figures 1(d) and 1(e)). Meanwhile, immunohistochemical staining also showed that MT-1/2 expression was relatively reduced mainly around the bronchial epithelia and areas of leukocyte infiltration in H3N2-infected mice (Figure 1(f)). Likewise, melatonin administration also upregulated the expressed intensity of MT-1/2 in H3N2-infected mice, especially around the bronchial epithelia (Figure 1(f)).

### 3.2. Melatonin Inhibited Influenza A- (H3N2-) Induced ALI and Pyroptosis

**Figure 2:**
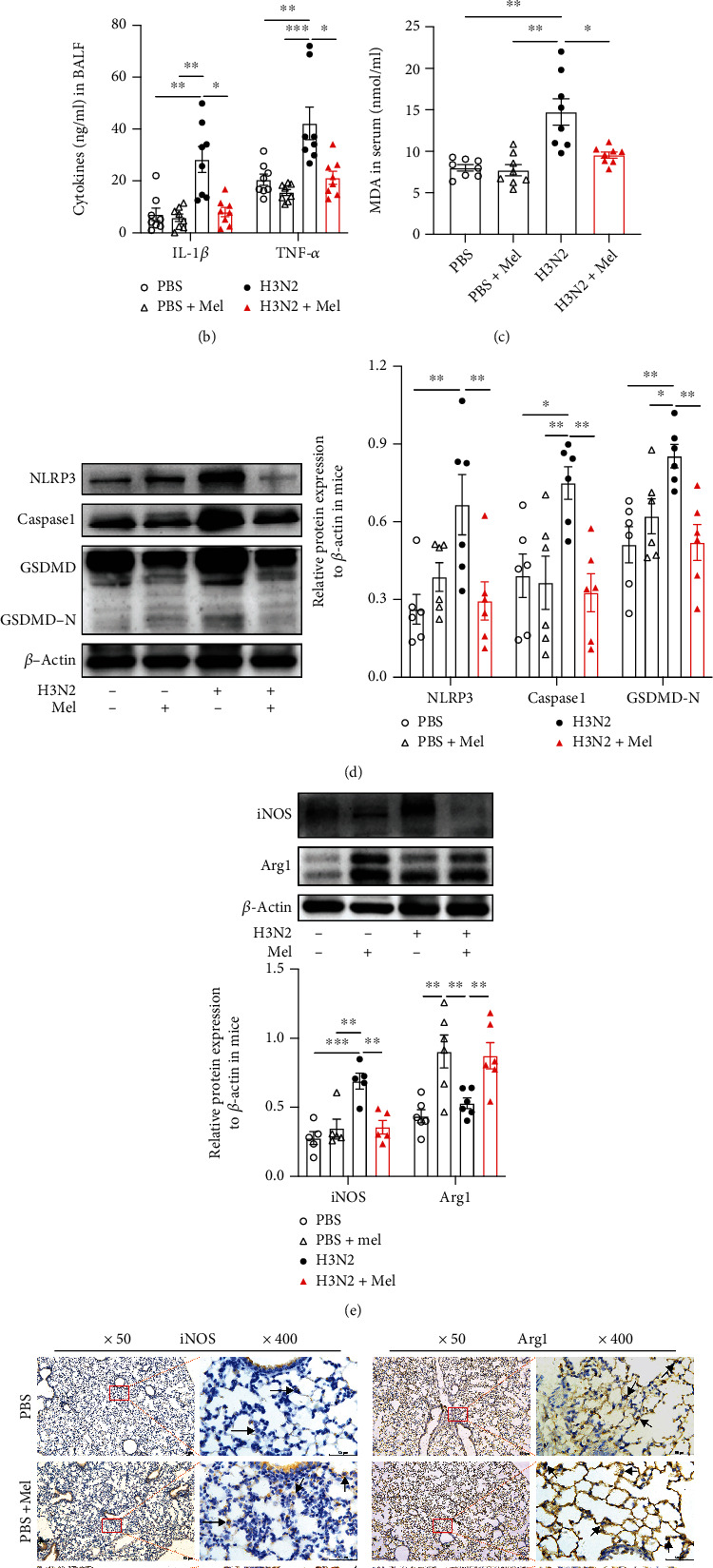
The protective effects of melatonin on ALI induced by influenza A (H3N2). (a) Representative bronchial and alveolar photomicrographs of murine lung tissues in H&E-stained sections as well as individual values of lung injury scores from the control (PBS) group, PBS+Mel group, H3N2 infection group, and H3N2+Mel group, bar 50 *μ*m (original magnification ×50, ×200). (b) Quantitative ELISA detection of IL-1*β* and TNF-*α* levels in BALF of mice. (c) Individual MDA contents in serum were detected using a MDA assay kit. Western blot analysis of the expression of NLRP3, Caspase1, and GSDMD-N (d) as well as iNOS and Arg1 (e) to *β*-actin in lung tissue homogenates. (f) Representative immunohistochemical images of lung tissues stained with M1 marker iNOS and M2 marker Arg1 as indicated by the brown staining (black arrows) from the control (PBS) group, PBS+Mel group, H3N2 infection group, and H3N2+Mel group, bar 50 *μ*m (original magnification ×50, ×400). Data expressed as mean ± SEM (*n* ≥ 3). ^∗^*p* < 0.05, ^∗∗^*p* < 0.01, and ^∗∗∗^*p* < 0.001 compared with influenza A- (H3N2-) infected mice.

**Figure 3:**
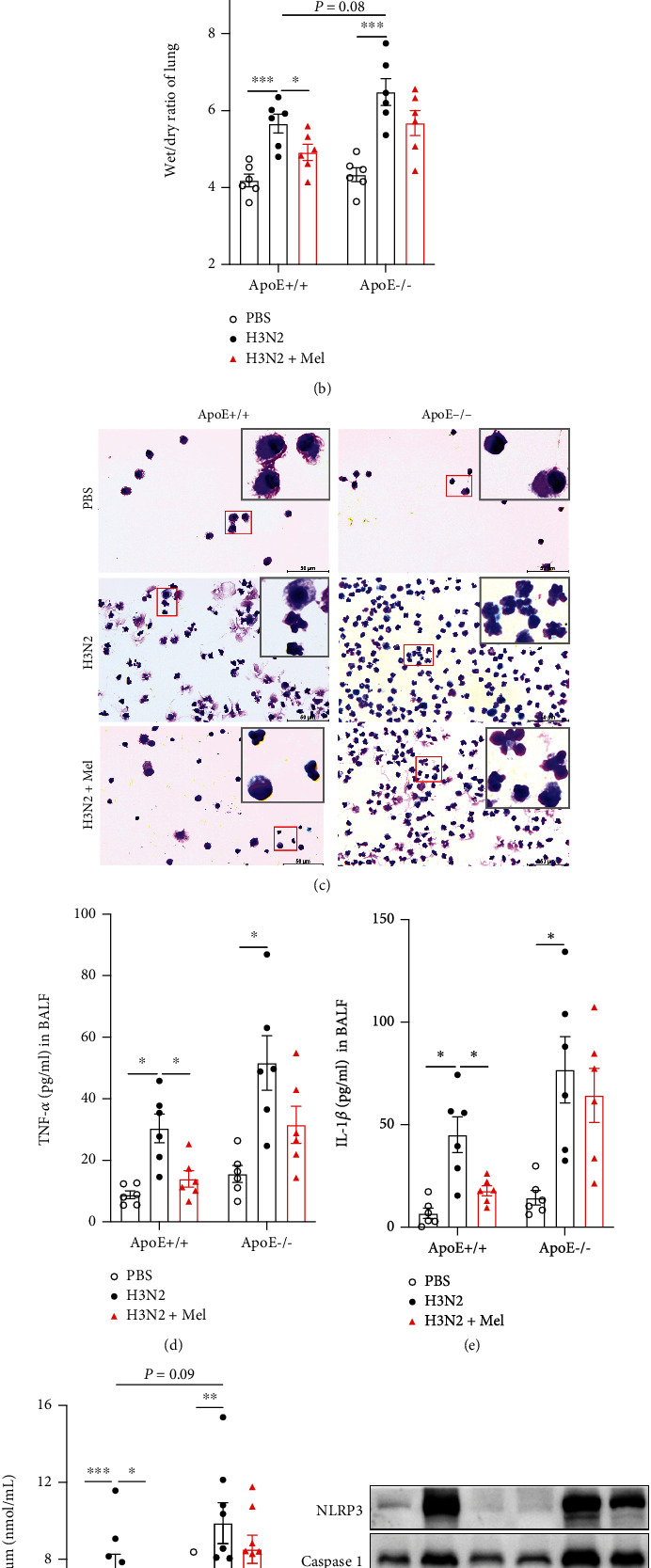
The protective impacts of melatonin in influenza A- (H3N2-) infected ApoE-/- mice. (a) Representative bronchial and alveolar photomicrographs of wild-type (WT) and ApoE-/- mouse lung tissues in H&E-stained sections as well as individual values of lung injury scores from the control (PBS) group, H3N2 infection group, and H3N2+Mel group, bar 50 *μ*m (original magnification ×50, ×200). (b) Representative lung morphology and individual values of wet/dry ratio measured from the ratio of wet lung weight to dry lung weight of WT and ApoE-/- mice from the control (PBS) group, H3N2 infection group, and H3N2+Mel group. (c) Wright-Giemsa staining of BAL cells in WT and ApoE-/- mice from the control (PBS) group, H3N2 infection group, and H3N2+Mel group, bar 50 *μ*m (original magnification × 400). Quantitative ELISA detection of TNF-*α* (d) and IL-1*β* (e) levels in BALF of WT and ApoE-/- mice. (f) Individual MDA contents in serum of WT and ApoE-/- mice. (g) Western blot analysis of the expression of NLRP3, Caspase1, and GSDMD-N to *β*-actin in lung tissue homogenate of WT and ApoE-/- mice. (h) Quantitative RT-PCR measurement of the relative mRNA level of IL-1*β* of WT and ApoE-/- mouse lung tissues. Data expressed as mean ± SEM (*n* ≥ 3). ^∗^*p* < 0.05, ^∗∗^*p* < 0.01, and ^∗∗∗^*p* < 0.001 compared with influenza A- (H3N2-) infected WT and ApoE-/- mice.

HE staining showed that H3N2 infection induced significant pulmonary destruction and leukocyte infiltration on day 7, whereas severe lung injury and fibrous changes happened on day 14 in lung tissues (Figure S1(b)). Therefore, we chose H3N2 infection for 7 consecutive days to establish the ALI mouse model (Figure S1(c)). Administration of melatonin significantly attenuated H3N2-induced ALI with the decreases of lung injury scores (Figure 2(a)) and also reduced the wet/dry ratio of the lung (Figure 3(b)), indicating inhibiting lung edema. Meanwhile, melatonin significantly decreased total leukocyte counting, especially neutrophils and macrophages (Figure S2 (d-f)). Moreover, compared to the H3N2 infection group, melatonin significantly decreased the levels of IL-1*β* and TNF-*α* in BALF (Figure 2(b)) and reversed H3N2-induced increases of MDA contents in the serum of mice (Figure 2(c)). Subsequently, melatonin significantly reduced H3N2-induced increases in the expression of NLRP3, Caspase1, and GSDMD-N protein (Figure 2(d)), indicating suppressing oxidative stress and pyroptosis in mice.

### 3.3. Melatonin Inhibited the M1 Polarization of Pulmonary Macrophages in Mice

**Figure 4:**
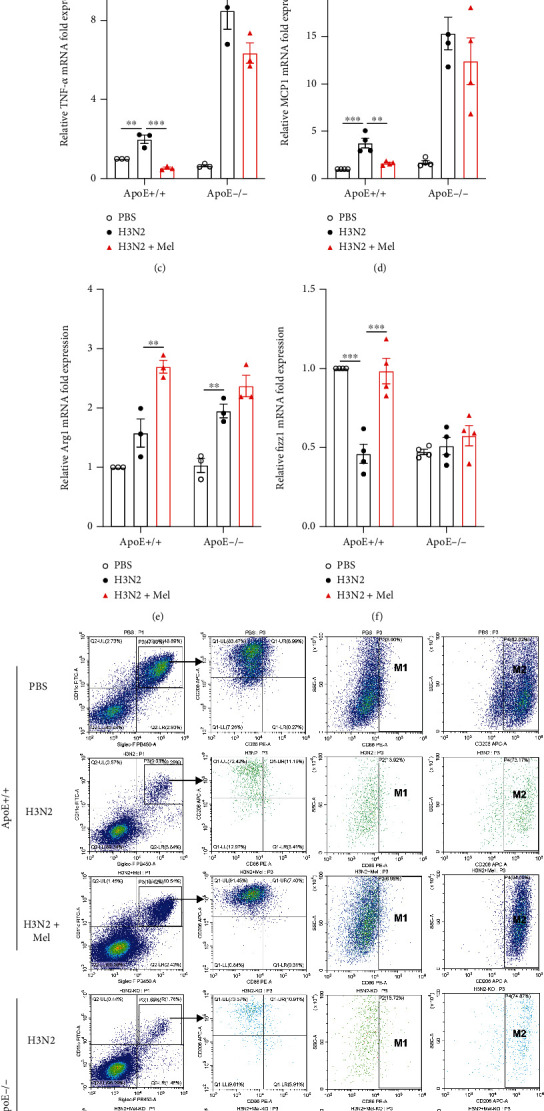
The blocking of the ApoE/LDLR pathway suppressed the regulation of macrophage polarization by melatonin. (a) Western blot images of the expression of ApoE, LDLR, iNOS, and Arg1 to *β*-actin in lung tissue homogenate of WT and ApoE-/- mice from the control (PBS) group, H3N2 infection group, and H3N2+Mel group. (b) Representative immunohistochemistry images of WT and ApoE-/- mouse lung tissues stained with iNOS and Arg1 as indicated by the brown staining (black arrows) from the control (PBS) group, H3N2 infection group, and H3N2+Mel group, bar 50 *μ*m (original magnification ×50, ×400). Quantitative RT-PCR measurements of the relative mRNA levels of TNF-*α* (c), MCP1 (d), Arg1 (e), and Fizz1 (f) in lung tissues of WT and ApoE-/- mice. (g) Gating strategy of flow cytometry analysis to identify alveolar macrophages (AMs) (CD45+Siglec-F+CD11c+) as well as the M1 (CD86+) and M2 (CD206+) phenotypes of AMs in BALF. Individual percentages of AMs (h) in total leukocytes of BALF, CD86+ AMs (i), and CD206+ AMs (j) in total AMs in WT and ApoE-/- mice from the control (PBS) group, H3N2 infection group, and H3N2+Mel group. Data expressed as mean ± SEM (*n* ≥ 3). ^∗^*p* < 0.05, ^∗∗^*p* < 0.01, and ^∗∗∗^*p* < 0.001 compared with influenza A- (H3N2-) infected mice.

Immunohistochemical staining showed that iNOS (M1 marker) expression was relatively increased around the areas of leukocyte infiltration of bronchial epithelia and alveolar walls in H3N2-infected mice (Figure 2(f)). After melatonin treatment, iNOS expressed intensity showed an obvious decrease, whereas Arg1 (M2 marker) expression showed a marked enhancement in H3N2-infected lung tissues (Figure 2(f)), as indicated in western blot analysis of iNOS and Arg1 of lung tissues (Figure 2(e)). Similarly, PCR analysis also showed that melatonin reversed H3N2-induced increases of M1 markers (IL-1*β*, TNF-*α*, and MCP1) and upregulated the mRNA expression of M2 markers (Arg1 and Fizz1) in H3N2-infected mice (Figures S2(a) and S2(b)). In order to further investigate the effects of melatonin on the polarization of pulmonary macrophages, alveolar macrophages (AMs) in BALF were defined by flow cytometry analysis based on the specific markers of M1 AMs (CD45+Siglec-F+CD11c+CD86+ population) and M2 AMs (CD45+Siglec-F+CD11c+CD206+ population). Flow cytometry analysis of BAL cells revealed that H3N2 infection significantly decreased the percentage of AMs, particularly CD206+ AMs, and increased CD86+ AMs (Figures 4(g)–4(j)). However, administration of melatonin increased the percentage of CD206+ AMs and decreased the percentage of CD86+ AMs in H3N2-infected wild-type (WT) mice (Figures 4(g)–4(j)). These results suggested that melatonin exerted anti-inflammatory effects by inhibiting the M1 polarization of pulmonary macrophages in H3N2-induced ALI.

### 3.4. Melatonin Inhibited H3N2-Induced M1 Polarization and Oxidative Injury of Raw264.7 Cells

**Figure 5:**
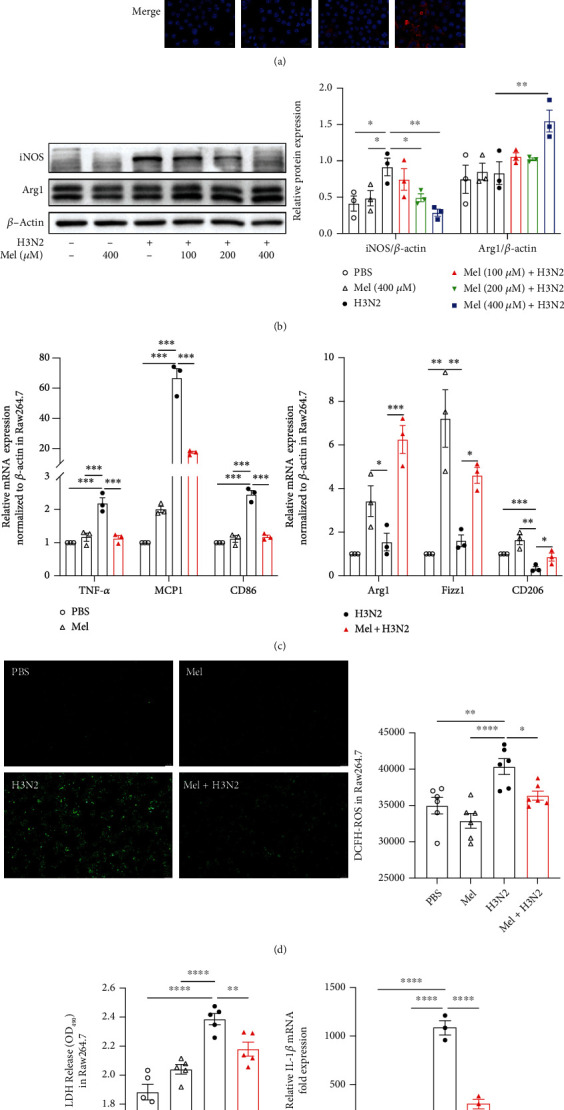
The effects of melatonin on macrophage polarization and oxidative injury in Raw264.7 cells. (a) Representative immunofluorescence images of iNOS (green) and Arg1 (red) expression in Raw264.7 cells infected by influenza A (H3N2) infection (MOI = 2, 12 h) with/without melatonin pretreatment (400 *μ*M, 3 h before H3N2 infection) (original magnification ×630). (b) Western blot analysis of the expression of iNOS and Arg1 to *β*-actin in Raw264.7 cells. (c) Quantitative RT-PCR measurements of the relative mRNA levels of TNF-*α*, MCP1, CD86, Arg1, Fizz1, and CD206 in Raw264.7 cells. (d) Representative images of DCF fluorescence (green) in Raw264.7 cells (original magnification ×50); quantitative ROS values were detected using a fluorescence plate reader. (e) The lactic dehydrogenase (LDH) released into the medium was assessed based on OD_490_ values of LDH release in Raw264.7 cells. (f) Quantitative RT-PCR measurement of the relative mRNA level of IL-1*β* in Raw264.7 cells. (g) Western blot analysis of the expression of NLRP3, Caspase1, and GSDMD-N to *β*-actin in Raw264.7 cells. Data expressed as mean ± SEM (*n* ≥ 3). ^∗^*p* < 0.05, ^∗∗^*p* < 0.01, and ^∗∗∗^*p* < 0.001 compared with influenza A- (H3N2-) infected Raw264.7 cells.

In vitro, after H3N2 infection, the morphology of Raw264.7 cells showed marked differentiation from circular toward spindle shapes (Figure S3(a)), indicating the M1 polarization of Raw264.7 cells. Immunofluorescence staining showed that melatonin significantly attenuated H3N2-induced enhancement of iNOS fluorescence intensity and upregulated Arg1 fluorescence intensity in H3N2-stimulated Raw264.7 cells (Figure 5(a)), in line with the results of western blot analysis (Figure 5(b)). Specifically, the gray value ratio of Arg1 and iNOS showed a significant elevation after melatonin prevention (Figure S3(b)), indicating the M2 polarization of Raw264.7 cells. Meanwhile, H3N2 infection promoted the mRNA expression of TNF-*α*, MCP1, and CD86, whereas it was inhibited by melatonin (Figure 5(c)). Melatonin also increased the mRNA expression of Arg1, Fizz1, and CD206 in H3N2-infected Raw264.7 cells (Figure 5(c)). Moreover, ROS burst was enhanced by H3N2 stimulation and inhibited by melatonin pretreatment in Raw264.7 cells (Figure 5(d)). And melatonin inhibited H3N2-induced increases in the protein expression of NLRP3, Caspase1, and GSDMD-N as well as IL-1*β* mRNA expression and LDH release (Figures 5(e)–5(g)). Altogether, these results suggested that melatonin inhibited the M1 polarization, ROS production, and pyroptosis of H3N2-infected Raw264.7 cells.

### 3.5. ApoE/LDLR Pathway Was Involved in the Protective Impacts of Melatonin

**Figure 6:**
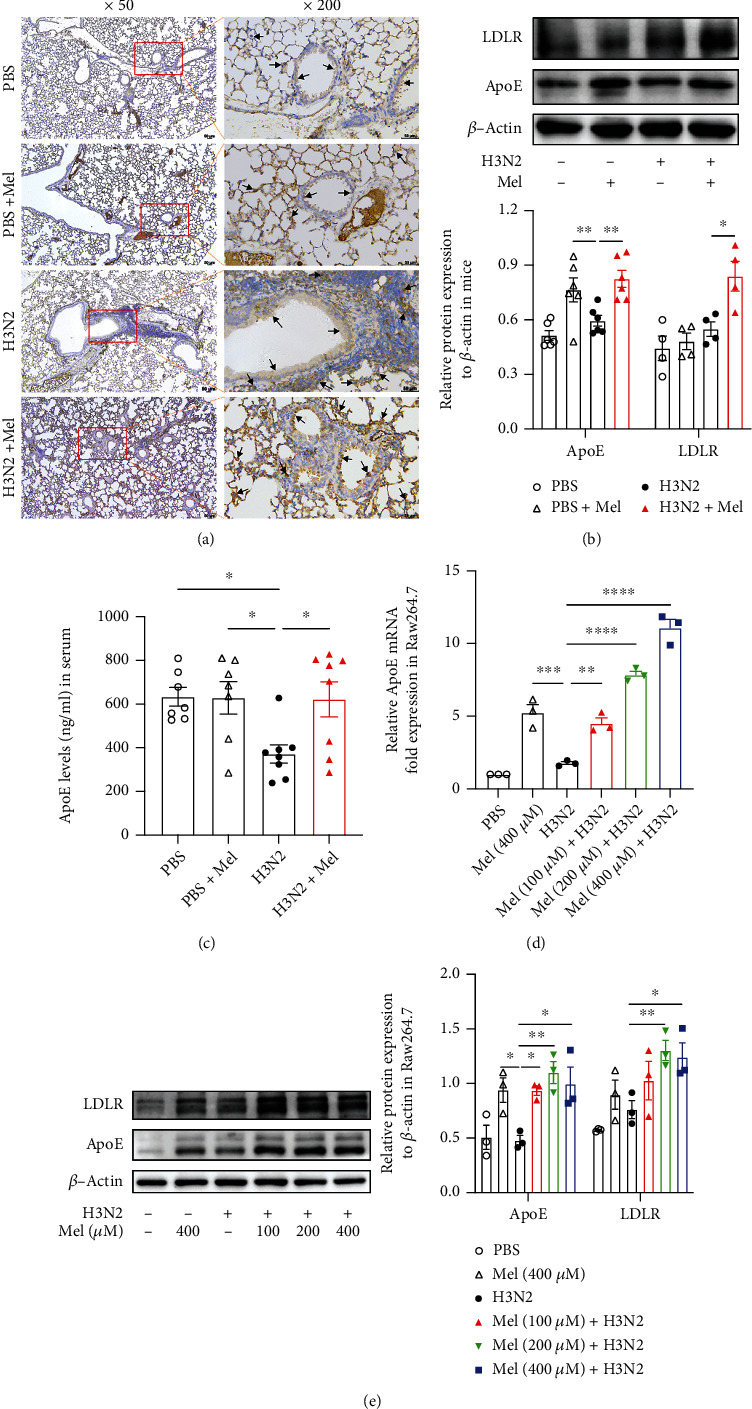
Melatonin promoted the activation of the ApoE/LDLR pathway in H3N2-induced ALI. (a) Representative immunohistochemical images of ApoE in lung tissues of wild-type (WT) mice as indicated by the brown staining (black arrows) from the control (PBS) group, PBS+Mel group, H3N2 infection group, and H3N2+Mel group, bar 50 *μ*m (original magnification ×50, ×200). (b) Western blot analysis of the expression of ApoE and LDLR to *β*-actin in lung tissue homogenate of WT mice. (c) Quantitative ELISA detection of ApoE levels in the serum from the control (PBS) group, PBS+Mel group, H3N2 infection group, and H3N2+Mel group. (d) Quantitative RT-PCR measurement of the relative mRNA level of ApoE in Raw264.7 cells infected with influenza A (H3N2) (MOI = 2, 12 h) with/without melatonin pretreatment (100 *μ*M, 200 *μ*M, or 400 *μ*M, 3 h before H3N2 infection). (e) Western blot analysis of the expression of ApoE and LDLR to *β*-actin in Raw264.7 cells. Data expressed as mean ± SEM (*n* ≥ 3). ^∗^*p* < 0.05, ^∗∗^*p* < 0.01, and ^∗∗∗^*p* < 0.001 compared with influenza A- (H3N2-) infected mice or Raw264.7 cells.

Recent evidence demonstrated that melatonin exerted anti-inflammation probably involved in the activation of ApoE signaling [31]. Immunohistochemical staining showed that ApoE expressed intensity was obviously enhanced around bronchial epithelia and alveolar walls after melatonin intervention in H3N2-infected mice (Figure 6(a)). And ApoE protein expression also showed an obvious increase after melatonin intervention accompanied by an increase of LDLR expression in H3N2-infected mice (Figure 6(b)). ELISA also demonstrated that serum ApoE levels significantly decreased after H3N2 infection, whereas they were upregulated after melatonin intervention (Figure 6(c)). Additionally, in H3N2-infected Raw264.7 cells, melatonin intervention also significantly upregulated ApoE mRNA expression (Figure 6(d)) and promoted the protein expression of ApoE and LDLR (Figure 6(e)). These results suggested that the ApoE/LDLR pathway was positively related with the protective impacts of melatonin.

### 3.6. ApoE Knockout Mostly Abrogated Anti-Inflammatory Impacts of Melatonin on Influenza A- (H3N2-) Infected Mice

To investigate whether melatonin inhibited H3N2-induced ALI in an ApoE-dependent manner, ApoE-/- mice were infected with H3N2 with melatonin intervention. The PCR analysis showed that ApoE mRNA expression was entirely lost in ApoE-/- mice (Figure S2(c)). H&E staining showed that H3N2-induced ALI was further aggravated in ApoE-/- mice compared to that of WT mice, mainly manifested in more severe leukocyte infiltration and hyaline membrane formation as well as alveolar septal thickening (Figure 3(a)). And lung morphology showed that H3N2 infection induced more obvious lung hyperaemia and edema in ApoE-/- mice compared to that of WT mice, as indicated in analysis of the wet/dry ratio of the lung (Figure 3(b)). However, melatonin administration failed to improve H3N2-induced ALI and edema of ApoE-/- mice (Figures 3(a) and 3(b)). BAL cell smearing and counting also showed that H3N2 infection further increased total leukocyte counting, especially neutrophils and macrophages in ApoE-/- mice, but no obvious reduction after melatonin intervention (Figure 3(c) and Figure S2(d-f)).

Moreover, melatonin did not reduce H3N2-induced increases in TNF-*α* and IL-1*β* levels of BALF as well as MDA contents of serum in ApoE-/- mice (Figures 3(d)–3(f)). Accordingly, melatonin failed to inhibit the protein expression of NLRP3, Caspase1, and GSDMD-N (Figure 3(g) and Figure S4(a)), as well as IL-1*β* mRNA expression in H3N2-infected ApoE-/- mice (Figure 3(h)), suggesting that the antioxidant and antipyroptosis ability of melatonin was almost lost in ApoE-/- mice.

### 3.7. ApoE Knockout Suppressed the Regulation of Melatonin on Macrophage Polarization in Mice

In H3N2-infected ApoE-/- mice, melatonin failed to increase ApoE and LDLR expression (Figure 4(a) and Figure S4(b)). There was a significant increase of iNOS expression in H3N2-infected ApoE-/- mice compared to that of WT mice. However, melatonin failed to inhibit iNOS expression and promote Arg1 expression in H3N2-infected ApoE-/- mice (Figure 4(a) and Figure S4(c)). Similarly, immunohistochemical staining showed that iNOS expressed intensity in areas of infiltration of leukocytes was obviously enhanced, and Arg1 expressed intensity was attenuated, whereas there were no obvious changes in expressed intensities of iNOS and Arg1 after melatonin intervention in H3N2-infected ApoE-/- mice (Figure 4(b)). Additionally, the PCR results indicated that melatonin inhibited H3N2-induced increases of mRNA expression of TNF-*α* and MCP1 and promoted mRNA expression of Arg1 and Fizz1 in H3N2-infected WT mice (Figures 4(c)–4(f)). Oppositely, in ApoE-/- mice, melatonin failed to do this (Figures 4(c)–4(f)). Similarly, flow cytometry analysis of BAL cells revealed that administration of melatonin had no significant effects on the percentage of AMs, especially CD86+ AMs and CD206+ AMs in H3N2-infected ApoE-/- mice (Figures 4(g)–4(j)). These results indicated that ApoE knockout mostly abrogated the regulatory impacts of melatonin on the polarization of pulmonary macrophages.

### 3.8. re-ApoE3 Promoted Influenza A- (H3N2-) Induced M1 BMDMs to M2 Polarization

**Figure 7:**
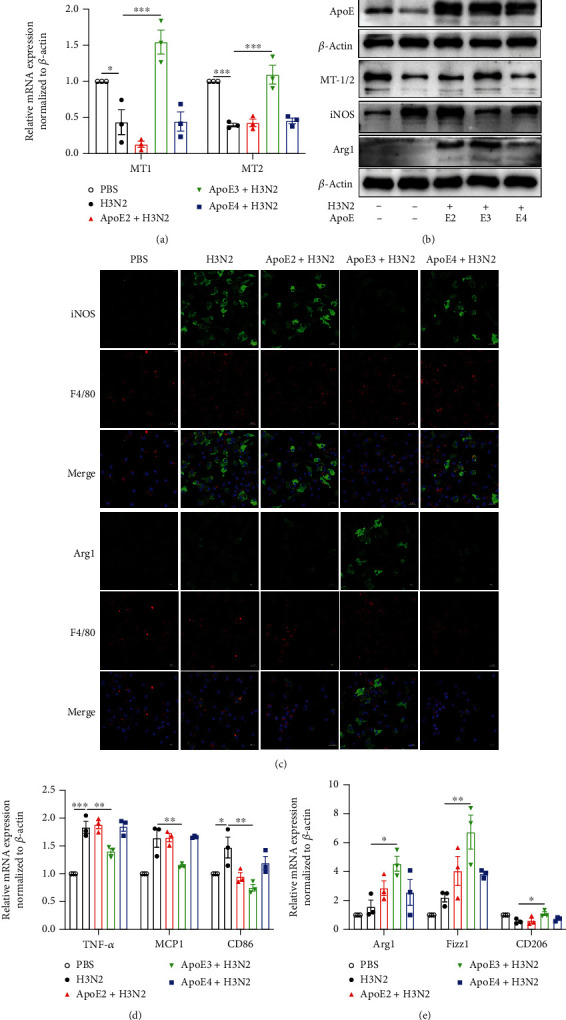
The effect of re-ApoE on the polarization of BMDMs and melatonin receptors. (a) Quantitative RT-PCR measurements of the relative mRNA levels of MT1 and MT2 in wild-type (WT) BMDMs infected by influenza A (H3N2) (MOI = 2, 12 h) with pretreatments of recombinant ApoE2, ApoE3, and ApoE4 (10 *μ*g/ml, 3 h before H3N2 infection). (b) Western blot images of the expression of ApoE, MT-1/2, iNOS, and Arg1 to *β*-actin in WT BMDMs. (c) Representative immunofluorescence images of F4/80 (red), iNOS (green), and Arg1 (green) expression in WT BMDMs (original magnification × 400). (d, e) Quantitative RT-PCR measurements of the relative mRNA levels of TNF-*α*, MCP1, CD86, Arg1, Fizz1, and CD206 in WT BMDMs. Data expressed as mean ± SEM (*n* ≥ 3). ^∗^*p* < 0.05, ^∗∗^*p* < 0.01, and ^∗∗∗^*p* < 0.001 compared with influenza A- (H3N2-) infected BMDMs.

To further explore the role of ApoE on macrophage polarization regulated by melatonin, recombinant ApoE proteins (re-ApoE2, re-ApoE3, and re-ApoE4) were added in H3N2-infected bone marrow-derived macrophages (BMDMs). Flow cytometry analysis showed that the purity of matured BMDMs reached over 95% (Figure S5). The PCR analysis showed that re-ApoE3 pretreatment significantly reversed H3N2-induced decreases in the mRNA expression of MT1 and MT2 as well as the protein expression of MT-1/2, but no effects after re-ApoE2 and re-ApoE4 pretreatment in H3N2-infected BMDMs (Figures 7(a) and 7(b) and Figure S6(b)), suggesting that ApoE3 may affect the secretion of melatonin via regulating the expression of melatonin receptors.

The morphology of BMDMs showed marked differentiation from original spindle shapes toward circular shapes after H3N2 stimulation, whereas it was reversed by re-ApoE3 intervention (Figure S6(a)). Western blot analysis indicated that ApoE expression significantly increased in H3N2-infected BMDMs with re-ApoE2 and re-ApoE3 pretreatments, but no significant increase on re-ApoE4 (Figure 7(b) and Figure S6(c)). And re-ApoE3 significantly reversed H3N2-induced increase of iNOS expression and promoted Arg1 expression (Figure 7(b) and Figure S6(d)). Likewise, immunofluorescence staining also showed that only re-ApoE3 significantly alleviated the fluorescence intensity of iNOS and enhanced that of Arg1 in H3N2-infected BMDMs (Figure 7(c)), indicating the M2 polarization of BMDMs by ApoE3. Additionally, re-ApoE3 significantly inhibited the mRNA expression of TNF-*α*, MCP1, and CD86 and increased the mRNA expression of Arg1, Fizz1, and CD206 in H3N2-infected BMDMs (Figures 7(d) and 7(e)). These results indicated that re-ApoE3 exerts potential anti-inflammatory impacts by modulating macrophage polarization.

### 3.9. Re-ApoE3 Enhanced the Regulatory Ability of Melatonin on Macrophage Polarization and Oxidative Injury

**Figure 8:**
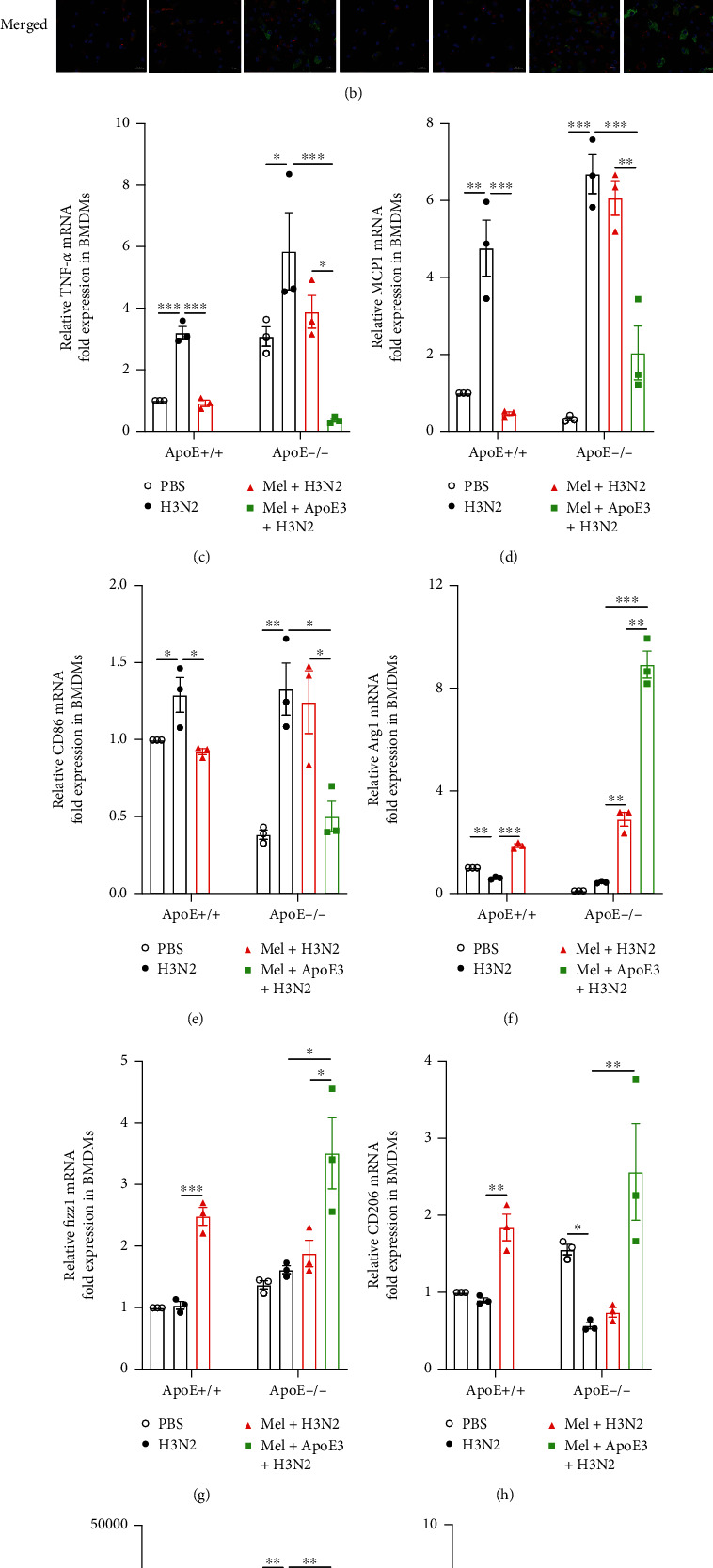
re-ApoE3 remedied the loss of regulatory ability of melatonin on macrophage polarization and oxidative injury. (a) Western blot images of the expression of ApoE, LDLR, iNOS, and Arg1 to *β*-actin in wild-type (WT) and ApoE-/- BMDMs infected by influenza A (H3N2) (MOI = 2, 12 h) with/without melatonin pretreatment or combined pretreatment of melatonin and recombinant ApoE3 (10 *μ*g/ml, 3 h before H3N2 infection). (b) Representative immunofluorescence images of F4/80 (red), iNOS (green), and Arg1 (green) expression in WT and ApoE-/- BMDMs (original magnification ×400). Quantitative RT-PCR measurement of the relative mRNA levels of TNF-*α* (c), MCP1 (d), CD86 (e), Arg1 (f), Fizz1 (g), CD206 (h), and IL-1*β* (j) in WT and ApoE-/- BMDMs. (i) Intracellular ROS levels were quantificationally detected by DCFH-DA using a fluorescence plate reader in WT and ApoE-/- BMDMs. (k) Western blot images of the expression of NLRP3, Caspase1, and GSDMD-N to *β*-actin in WT and ApoE-/- BMDMs. (l) The lactic dehydrogenase (LDH) released into the medium was assessed based on OD_490_ values of LDH release in WT and ApoE-/- BMDMs. Data expressed as mean ± SEM (*n* ≥ 3). ^∗^*p* < 0.05, ^∗∗^*p* < 0.01, and ^∗∗∗^*p* < 0.001 compared with influenza A- (H3N2-) infected WT or ApoE-/- BMDMs.

In H3N2-infected ApoE-/- BMDMs, melatonin failed to promote ApoE, LDLR, and Arg1 expression and inhibit iNOS expression (Figure 8(a) and Figure S7(a, b)). However, melatonin combined with re-ApoE3 significantly upregulated the protein levels of ApoE, LDLR, and Arg1 and inhibited H3N2-induced increase of iNOS expression compared to H3N2-infected ApoE-/- BMDMs (Figure 8(a) and Figure S7(a, b)), as shown by immunofluorescence staining of iNOS and Arg1 in BMDMs (Figure 8(b)). Likewise, melatonin and re-ApoE3 cotreatment further effectively inhibited the mRNA expression of TNF-*α*, MCP1, and CD86 and promoted the mRNA expression of Arg1, Fizz1, and CD206 compared to single melatonin intervention in H3N2-infected ApoE-/- BMDMs (Figures 8(c)–8(h)). Moreover, the morphological images of BMDMs also showed marked differentiation from circular shapes toward spindle shapes after melatonin and re-ApoE3 cotreatment in H3N2-infected ApoE-/- BMDMs (Figure S7(c)).

Next, in H3N2-infected ApoE-/- BMDMs, melatonin failed to downregulate H3N2-induced increases of ROS levels (Figure 8(i)) and also did not inhibit the protein expression of NLRP3, Caspase1, and GSDMD-N (Figure 8(k) and Figure S7(d)). There were also no significant changes in IL-1*β* mRNA expression and LDH release after melatonin intervention in H3N2-infected ApoE-/- BMDMs (Figures 8(j) and 8(l)). Further assessing, we found that melatonin combined with re-ApoE3 significantly decreased ROS levels (Figure 8(i)) and inhibited the protein expression of NLRP3, Caspase1, and GSDMD-N as well as the IL-1*β* mRNA expression and LDH release in H3N2-infected ApoE-/- BMDMs (Figures 8(j)–8(l) and Figure S7(d)). These results demonstrated that exogenous re-ApoE3 enhanced the beneficial effects of melatonin, and the activation of the ApoE/LDLR pathway improved the modulation of melatonin on macrophage polarization, oxidative stress, and pyroptosis.

## 4. Discussion

**Figure 9:**
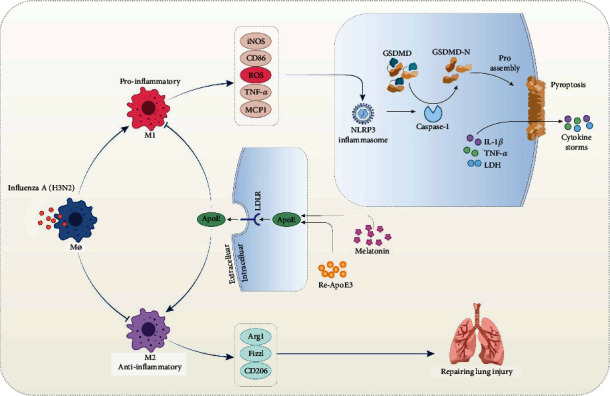
Schematic mechanisms underlying the regulatory effect of melatonin on influenza A- (H3N2-) induced acute lung injury.

In the present study, we put forward novel insights into the regulatory role of melatonin-ApoE/LDLR axis in macrophage polarization, oxidative stress, and pyroptosis and confirmed melatonin as a potential therapeutic agent in influenza virus-induced ALI, as indicated in Figure 9. Specifically, we proved that melatonin significantly attenuated influenza A- (H3N2-) induced ALI with the activation of the ApoE/LDLR pathway. ApoE knockout almost abrogated the protective impacts of melatonin on H3N2-induced ALI; re-ApoE3 inhibited H3N2-induced M1 polarization of BMDMs and inflammatory responses. Furthermore, melatonin and re-ApoE3 cotreatment reversed damaged effects induced by ApoE knockout, effectively switched macrophage polarization from M1 to M2 phenotype, and inhibited ROS production and pyroptosis. Taken together, melatonin suppressed macrophage M1 polarization and ROS-mediated pyroptosis via activating the ApoE/LDLR pathway in ALI. The conclusion provided new direct evidence that melatonin exerted the anti-inflammation and antioxidation in an ApoE-dependent manner.

In view of increasing global influenza and COVID-19 pandemics, more studies are devoted to elucidating the pathophysiology of ALI induced by virus infection for further developing the effective therapeutic agents [32, 33]. Particularly, relative studies indicated that the clinical and pathogenic features of SARS-CoV-2 infection had many parallels with influenza [34]. Virus-damaged epithelial cells can recruit a series of immune cells, especially macrophages, which induce the cascading amplification of inflammatory responses and the damage of lung structures [35]. Specially, at least three types of macrophages exist in lung tissues: bronchial macrophages, interstitial macrophages (IMs), and alveolar macrophages (AMs). Therein, AMs in the alveolar lumen form 90-95% of the cellular contents at homeostasis [36]. In ALI, monocytes recruited into the lung can differentiate into AMs. Under mild influenza A virus (IAV) infection, AMs can exert protective impacts through phagocytizing apoptotic epithelial cells [37]. With the exacerbation of IAV infection, the phagocytic capacity of AMs is decreased; oppositely, AMs may phagocytize IAV and assist the replication of progeny virus so as to infect surrounding cells [38]. Therefore, AMs also are regarded as a vehicle for virus dissemination. Moreover, IAV infection also induced a conversion of AMs toward M1 phenotype with an increase of iNOS expression [39]. And IAV-infected M2 BMDMs tended to polarize into M1 phenotype with excessive expression of iNOS and TNF-*α* [40]. Our results also showed that the M1 pulmonary macrophages were predominant in H3N2-induced ALI.

Studies have clarified that melatonin influenced multiple physiological functions of macrophages from host defenses to immune disorders, modulating inflammation and oxidation-antioxidant system [26, 41]. In a stress-induced inflammation model, melatonin switched macrophage polarization from M1 to M2 phenotype with increases of the M2 marker Arg1 and MRC1 expression and inhibited inflammatory injuries [42]. And in PM_2.5_-induced atherosclerosis, melatonin effectively alleviated PM_2.5_-induced oxidative damage of the aorta and atherosclerotic plaque formation via inhibiting macrophage M1 polarization and NOX2-mediated oxidative stress [43]. These studies demonstrate that melatonin can inhibit the M1 polarization of macrophages and oxidative stress. Accordingly, we firstly confirmed that melatonin mainly targeted pulmonary macrophages to exert the protective impacts in H3N2-infected mice. Mechanistically, melatonin inhibited H3N2-induced M1 polarization of pulmonary macrophages and oxidative stress.

With unbridled hyperinflammatory reactions, influenza virus infection will also lead to pyroptosis, a kind of programmed necrotic cell death [33]. Pyroptosis commonly relies on the gasdermin family members to cause cell rupturing and the formation of membrane pores. And GSDMD is considered as the real executioner of pyroptosis which is commonly cleaved to expose the N-terminal domains in a caspase1-dependent manner following NLRP3 inflammasome assembly [44]. Recent studies pointed that melatonin attenuated LPS-induced pyroptosis by inhibiting the NLRP3/GSDMD pathway which was primarily activated by ROS [29, 45]. Generally speaking, ROS are also regarded as biomarkers of M1 macrophage polarization [9]. Therefore, we can consider that the M1 polarization of macrophages promotes the occurrence of pyroptosis via activating the ROS-mediated NLRP3/GSDMD pathway. Consistently, a recent study proved that the M2 polarization of AMs alleviated LPS-induced lung pyroptosis via downregulating the Caspase1/GSDMD pathway [46]. As indicated in our results, melatonin inhibited ROS-driven activation of the NLRP3/GSDMD pathway via switching macrophage polarization from M1 to M2 phenotype.

Emerging evidence increasingly recognized that ApoE protein played a protective role in the development of lung diseases based on their ability to regulate inflammation and oxidative stress [15]. Previous studies indicated that ApoE-/- mice showed more severe pulmonary toxicity and neutrophil infiltration in the ALI model [47, 48], whereas administration of COG1410, an ApoE mimetic peptide, inhibited LPS-induced increases of alveolar neutrophils and macrophages [49]. And ApoE-/- mice transplanted with ApoE-expressed bone marrow showed increased plasma levels of IL-1RA (M2 Marker), and peritoneal macrophages of transplanted mice were also polarized into the M2 phenotype with increases of IL-1RA and CD206 levels [14]. Moreover, ApoE also had positive antioxidant effects, in a spinal cord injury mouse model; exogenous ApoE administration significantly improved oxidative stress and neural function via Nrf2/HO-1 signaling [50]. These studies indicated that ApoE can be considered as an anti-inflammatory and antioxidant protein with an ability of regulating macrophage polarization. Additionally, a recent study demonstrated that melatonin decreased ROS levels and caspase activity by upregulating ApoE expression in oxygen and glucose deprivation-reoxygenation- (OGD-R-) stimulated endothelial cells [31]. Particularly, melatonin and ApoE are all highly expressed in brains so that potential interaction may be existing between them. In our results, ApoE knockout almost abrogated the positive effects of melatonin on ALI, and exogenous ApoE3 remedied the protective impacts of melatonin. Therefore, we considered that melatonin exerted anti-inflammation and antioxidation in an ApoE-dependent manner. It is a completely new application area for the modulation of melatonin on macrophage polarization, oxidative stress, and pyroptosis via the ApoE/LDLR pathway.

Specifically, human ApoE is polymorphic with three variants that encodes different amino acids in codons 112 and 158: cysteine at both sites for ApoE2, arginine at both sites for ApoE4, and respectively, cysteine and arginine for ApoE3 [16]. This variation greatly modifies ApoE protein functions and causes minimal binding activity with LDLR in ApoE2. Accumulated ApoE2 may induce dysbetalipoproteinaemia and accelerate aging [51]. And the variation in ApoE4 may cause the loss of antioxidant ability and become the strongest risk factor of Alzheimer's disease (AD) [52]. ApoE3 is the one of the highest frequencies and accounts for 65-70% of total ApoE [53]. ApoE3 possesses the requisite lipid-binding ability and higher affinity with LDLR [54]. Moreover, the cysteine residues of ApoE3 drive the covalent binding with 4-hydroxynonenal (HNE) so as to inhibit lipid peroxidation and inflammation [44]. Studies indicated that human ApoE3-knockin mice decreased the levels of TNF-*α* and IL-1*β* and elevated the survival compared to those of ApoE4-knockin mice in the caecal ligation and puncture model [55]. Interestingly, in our results, re-ApoE3 switched H3N2-induced M1 BMDMs toward the M2 polarization and also increased the expression of melatonin receptors; however, re-ApoE2 and re-ApoE4 failed to do this. These results indicated a potential synergetic protective impact between melatonin and ApoE3. And re-ApoE3 also enhanced the regulated ability of melatonin on macrophage polarization, oxidative stress, and pyroptosis, which further proved that melatonin attenuated H3N2-induced ALI in an ApoE-dependent manner.

## 5. Conclusion

In this study, we have provided strong direct evidence for the first time that melatonin attenuated influenza A- (H3N2-) induced ALI by inhibiting macrophage M1 polarization and ROS-mediated pyroptosis via activating the ApoE/LDLR pathway. We found that re-ApoE3 exerted the positive protective impacts by promoting H3N2-induced M1 BMDMs toward M2 polarization and also enhanced the anti-inflammatory and antioxidant abilities of melatonin, indicating that melatonin-ApoE/LDLR axis may serve as a novel intervention signal for treating influenza A-induced ALI. Meanwhile, this study also provided a potential clue for the therapy of ARDS induced by the novel coronavirus SARS-CoV-2.

## Data Availability

The data that support the findings of this study are available from the corresponding authors upon reasonable request.

## Supplementary Materials

Supplementary Materials Supplementary materials associated with this article have been uploaded into Supplementary files. Supplementary Table 1: list of primer sequences used for RT-PCR analysis. Supplementary Figure S1: (a) the effect on the viability of Raw264.7 cells by influenza A (H3N2) with different multiplicities of infection (MOI = 0, 0.5, 1, 2, 4, 6, and 8). (b) Representative bronchial and alveolar photomicrographs of murine lung tissues in H&E-stained sections from mice infected with influenza A (H3N2) (50 *μ*l, 100 plaque forming units (PFUs)) (0 days, 4 days, 7 days, and 14 days), bar 50 *μ*m (original magnification ×50, ×200). (c) Protocol of establishing acute lung injury mouse model (mice were instilled intratracheally with H3N2 (50 *μ*l, 100 PFUs) on day 0 and day 3). From day 0, mice were injected intraperitoneally with melatonin (Mel) (30 mg/kg, dissolved in PBS containing 5% DMSO) (stated purity ≥ 98%, M5250, Sigma-Aldrich, USA) at daily 18:00 for 7 consecutive days. Data expressed as mean ± SEM (*n* ≥ 3). ^∗^*p* < 0.05, ^∗∗^*p* < 0.01, and ^∗∗∗^*p* < 0.001 compared with influenza A- (H3N2-) infected Raw264.7 cells. Supplementary Figure S2: (a, b) quantitative RT-PCR measurements of the relative mRNA levels of IL-1*β*, TNF-*α*, MCP1, Arg1, and Fizz1 in lung tissues of wild-type (WT) mice. (c) Quantitative RT-PCR measurement of the relative mRNA level of ApoE in lung tissues of wild-type (WT) or ApoE-/- mice. Individual and mean numbers of total cells (d), neutrophils (e), and macrophages (f) in BALF of WT and ApoE-/- mice from the control (PBS) group, H3N2 infection group, and H3N2+Mel group. Data expressed as mean ± SEM (*n* ≥ 3). ^∗^*p* < 0.05, ^∗∗^*p* < 0.01, and ^∗∗∗^*p* < 0.001 compared with influenza A- (H3N2-) infected WT and ApoE-/- mice. Supplementary Figure S3: (a) the cell morphology of Raw264.7 cells infected by influenza A (H3N2) (MOI = 2, 12 h) with/without melatonin pretreatment (400 *μ*M, 3 h before H3N2 infection) (original magnification ×200). (b) Relative ratio of Arg1 and iNOS was measured from the ratio of gray values of Arg1 and iNOS protein expression in Raw264.7 cells infected by influenza A (H3N2) (MOI = 2, 12 h) with/without melatonin pretreatment (100 *μ*M, 200 *μ*M, or 400 *μ*M, 3 h before H3N2 infection). Data expressed as mean ± SEM (*n* ≥ 3). ^∗^*p* < 0.05, ^∗∗^*p* < 0.01, and ^∗∗∗^*p* < 0.001 compared with influenza A- (H3N2-) infected Raw264.7 cells. Supplementary Figure S4: western blot analysis of the protein expression of NLRP3, Caspase1, and GSDMD-N (a); ApoE and LDLR (b); and iNOS and Arg1 (c) to *β*-actin in lung tissue homogenate of WT and ApoE-/- mice from the control (PBS) group, H3N2 infection group, and H3N2+Mel group. Data expressed as mean ± SEM (*n* ≥ 3). ^∗^*p* < 0.05, ^∗∗^*p* < 0.01, and ^∗∗∗^*p* < 0.001 compared with influenza A- (H3N2-) infected WT and ApoE-/- mice. Supplementary Figure S5: the purity analysis of murine bone marrow-derived macrophages (BMDMs). After culturing for 7 days, gating strategy of flow cytometry analysis was conducted to determine the purity of mature BMDMs (CD45+F4/80+CD11b+ population). Supplementary Figure S6: (a) the cell morphological images of WT murine BMDMs infected by influenza A (H3N2) (MOI = 2, 12 h) with pretreatments of recombinant ApoE2, ApoE3, and ApoE4 (10 *μ*g/ml, 3 h before H3N2 infection) (original magnification ×200). Western blot analysis of the expression of melatonin receptors 1/2 (MT-1/2) (b), ApoE (c), iNOS, and Arg1 (d) to *β*-actin in BMDMs. Data expressed as mean ± SEM (*n* ≥ 3). ^∗^*p* < 0.05, ^∗∗^*p* < 0.01, and ^∗∗∗^*p* < 0.001 compared with influenza A- (H3N2-) infected WT BMDMs. Supplementary Figure S7: Figure S7: western blot analysis of the protein expression of ApoE and LDLR (a), Arg1 and iNOS (b), and NLRP3, Caspase1, and GSDMD-N (d) to *β*-actin in WT and ApoE-/- BMDMs infected by influenza A (H3N2) (MOI = 2, 12 h) with/without melatonin pretreatment or combined pretreatment of melatonin and recombinant ApoE3 (10 *μ*g/ml, 3 h before H3N2 infection). (c) The cell morphological images of in WT and ApoE-/- BMDMs (original magnification ×200). Data expressed as mean ± SEM (*n* ≥ 3). ^∗^*p* < 0.05, ^∗∗^*p* < 0.01, and ^∗∗∗^*p* < 0.001 compared with influenza A- (H3N2-) infected WT and ApoE-/- BMDMs.
