# Supplementary material for: Melatonin Suppresses Macrophage M1 Polarization and ROS-Mediated Pyroptosis via Activating ApoE/LDLR Pathway in Influenza A-Induced Acute Lung Injury
Source: Oxid Med Cell Longev. 2022 Nov 15;2022:2520348. doi: 10.1155/2022/2520348 (PMC9681554; doi:10.1155/2022/2520348)
Supplement: Supplementary Materials — associated with this article have been uploaded into Supplementary files. Supplementary Table 1: list of primer sequences used for RT-PCR analysis. Supplementary Figure S1: (a) the effect on the viability of Raw264.7 cells by influenza A (H3N2) with different multiplicities of infection (MOI = 0, 0.5, 1, 2, 4, 6, and 8). (b) Representative bronchial and alveolar photomicrographs of murine lung tissues in H&E-stained sections from mice infected with influenza A (H3N2) (50 μl, 100 plaque forming units (PFUs)) (0 days, 4 days, 7 days, and 14 days), bar 50 μm (original magnification ×50, ×200). (c) Protocol of establishing acute lung injury mouse model (mice were instilled intratracheally with H3N2 (50 μl, 100 PFUs) on day 0 and day 3). From day 0, mice were injected intraperitoneally with melatonin (Mel) (30 mg/kg, dissolved in PBS containing 5% DMSO) (stated purity ≥ 98%, M5250, Sigma-Aldrich, USA) at daily 18:00 for 7 consecutive days. Data expressed as mean ± SEM (n ≥ 3). ∗p < 0.05, ∗∗p < 0.01, and ∗∗∗p < 0.001 compared with influenza A- (H3N2-) infected Raw264.7 cells. Supplementary Figure S2: (a, b) quantitative RT-PCR measurements of the relative mRNA levels of IL-1β, TNF-α, MCP1, Arg1, and Fizz1 in lung tissues of wild-type (WT) mice. (c) Quantitative RT-PCR measurement of the relative mRNA level of ApoE in lung tissues of wild-type (WT) or ApoE-/- mice. Individual and mean numbers of total cells (d), neutrophils (e), and macrophages (f) in BALF of WT and ApoE-/- mice from the control (PBS) group, H3N2 infection group, and H3N2+Mel group. Data expressed as mean ± SEM (n ≥ 3). ∗p < 0.05, ∗∗p < 0.01, and ∗∗∗p < 0.001 compared with influenza A- (H3N2-) infected WT and ApoE-/- mice. Supplementary Figure S3: (a) the cell morphology of Raw264.7 cells infected by influenza A (H3N2) (MOI = 2, 12 h) with/without melatonin pretreatment (400 μM, 3 h before H3N2 infection) (original magnification ×200). (b) Relative ratio of Arg1 and iNOS was measure [file 2520348.f1.zip › Table S1.doc]

**Supplementary Table S1**

**Primer sequences used for RT-PCR analysis**

| **Gene** | **Forward Primer** | **Reverse Primer** |
| --- | --- | --- |
| Mouse-β-actin | CATTGCTGACAGGATGCAGAAGG | TGCTGGAAGGTGGACAGTGAGG |
| Mouse-IL-1β | TGGACCTTCCAGGATGAGGACA | GTTCATCTCGGAGCCTGTAGTG |
| Mouse-TNF-α | GGTGCCTATGTCTCAGCCTCTT | GCCATAGAACTGATGAGAGGGAG |
| Mouse-ApoE | GCCGAACACAAGAAGCTGGAAG | GCCTTTACTTCCGTCATAGTGTC |
| Mouse-MCP1 | CCAATGAGTAGGCTGGAGAGCT | GTCTGGACCCATTCCTTCTTGG |
| Mouse-Arg1 | ACAAGACAGGGCTCCTTTCA | AGCAAGCCAAGGTTAAAGCC |
| Mouse-Fizz1 | ATCGTGGAGAATAAGGTCAAGG | TTGACACTAGTGCAAGAGAGAG |
| Mouse-CD86 | ACGTATTGGAAGGAGATTACAGCT | TCTGTCAGCGTTACTATCCCGC |
| Mouse-CD206 | GTTCACCTGGAGTGATGGTTCTC | AGGACATGCCAGGGTCACCTTT |
| Mouse-MT1 | GGAGGGTGAAACCTGACGAC | CCCAGCAAATGGCAAAGAGG |
| Mouse-MT2 | GGCTCCGTCTTCAACATCACC | GCAGAAGGACCAGCAGGGTG |
